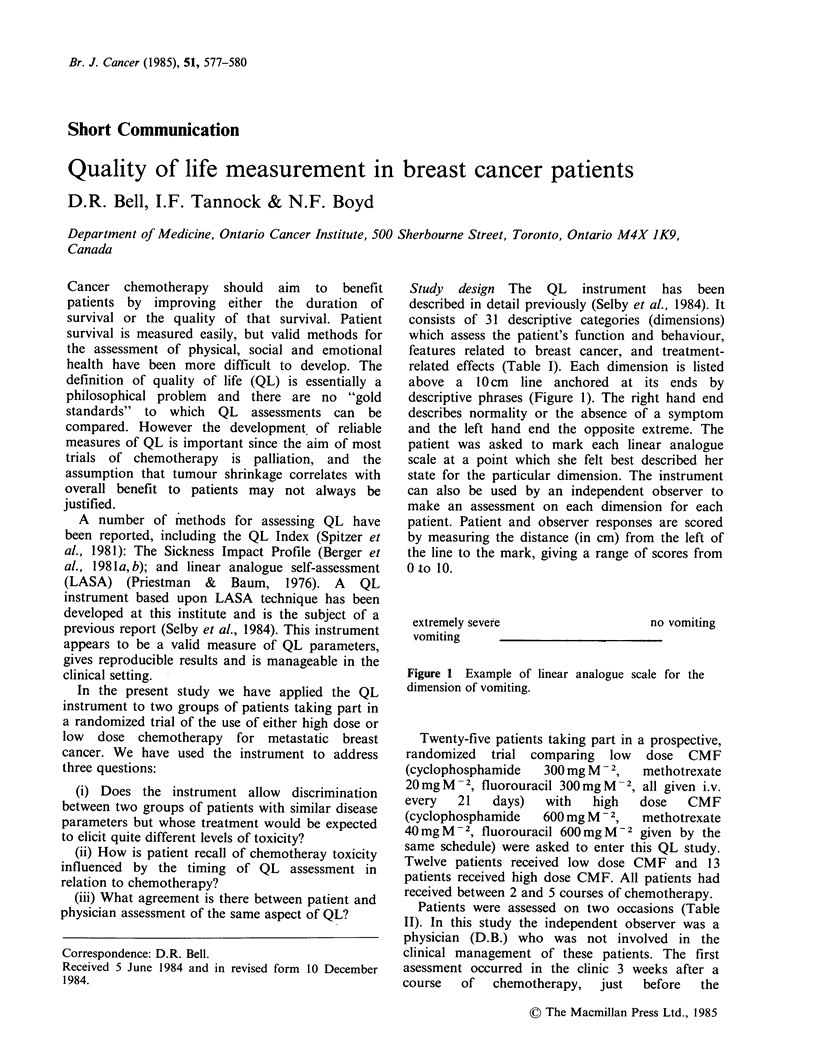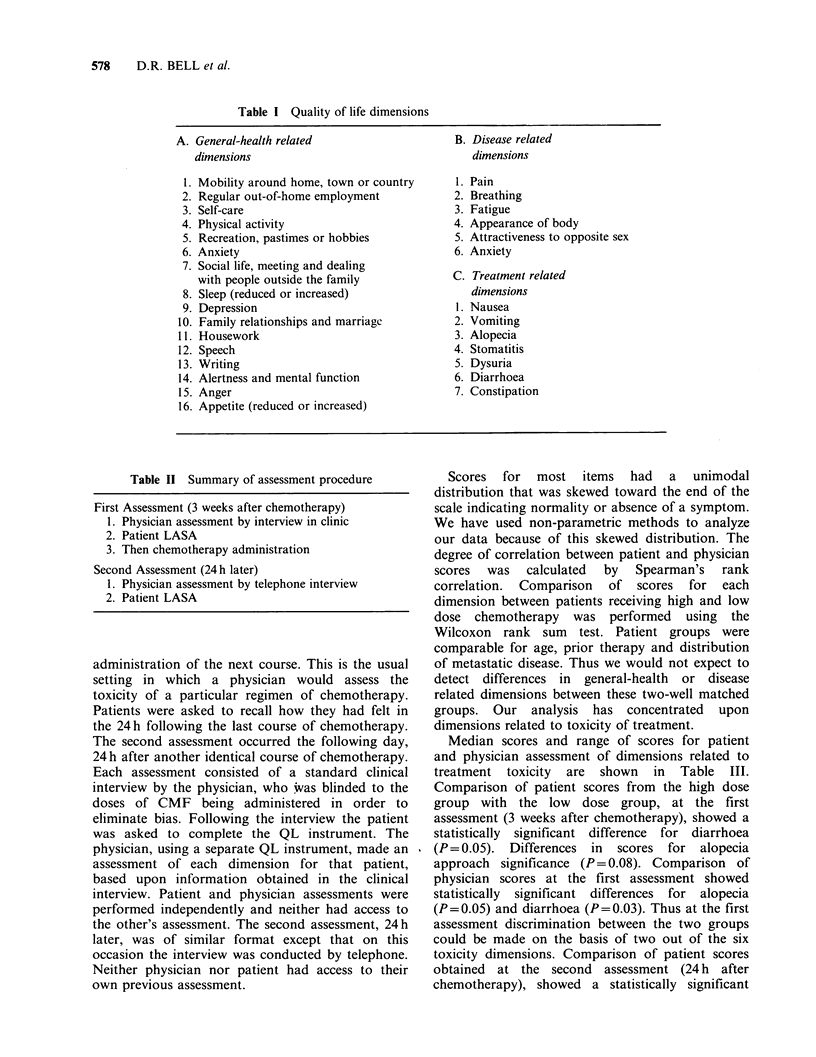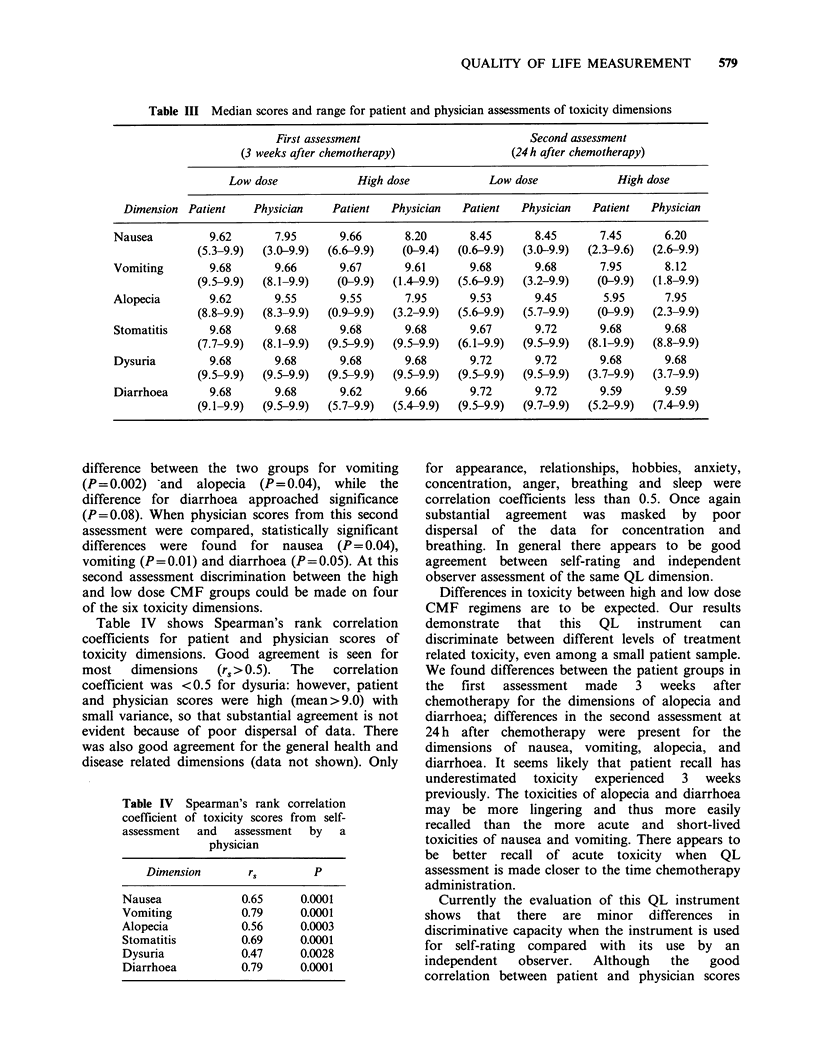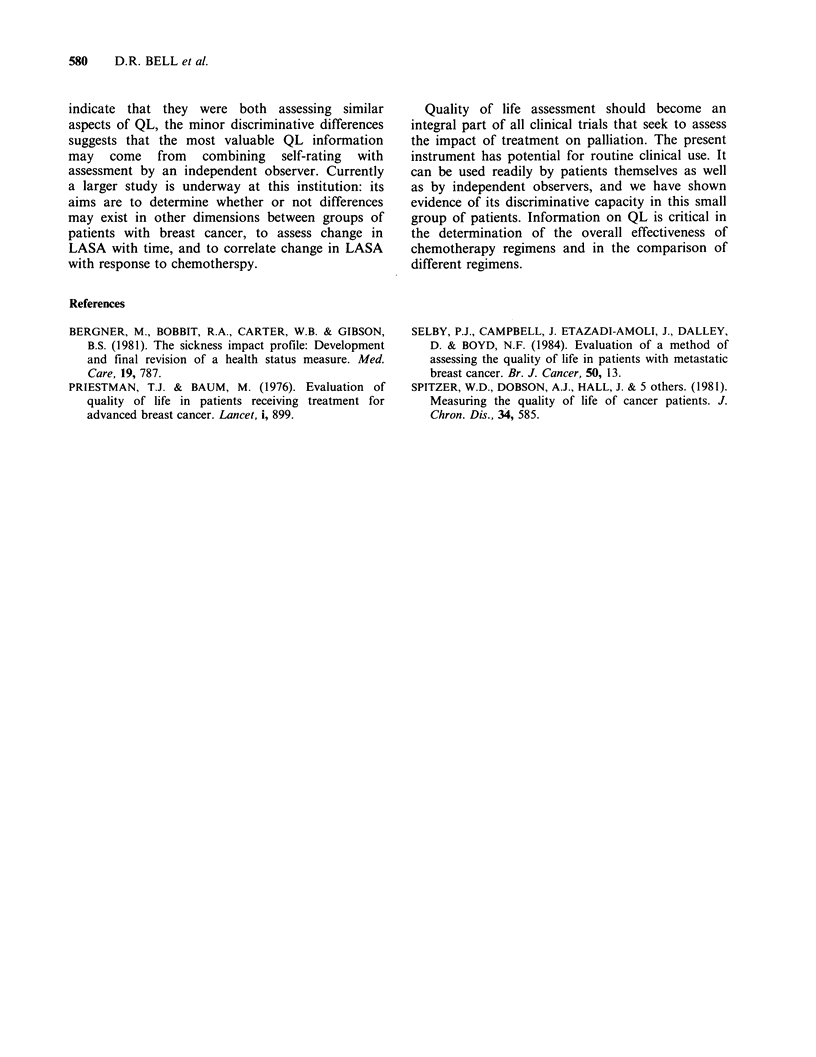# Quality of life measurement in breast cancer patients.

**DOI:** 10.1038/bjc.1985.81

**Published:** 1985-04

**Authors:** D. R. Bell, I. F. Tannock, N. F. Boyd


*Br. J. Cancer* (1985), **51,** 577–580

### Short Communication

### Quality of life measurement in breast cancer patients

D.R. Bell, I.F. Tannock & N.F. Boyd

*Department of Medicine, Ontario Cancer Institute, 500 Sherbourne Street, Toronto, Ontario M4X 1K9, Canada*

Cancer chemotherapy should aim to benefit patients by improving either the duration of survival or the quality of that survival. Patient survival is measured easily, but valid methods for the assessment of physical, social and emotional health have been more difficult to develop. The definition of quality of life (QL) is essentially a philosophical problem and there are no "gold standards" to which QL assessments can be compared. However the development of reliable measures of QL is important since the aim of most trials of chemotherapy is palliation, and the assumption that tumour shrinkage correlates with overall benefit to patients may not always be justified.

A number of methods for assessing QL have been reported, including the QL Index (Spitzer *et al.*, 1981): The Sickness Impact Profile (Berger *et al.*, 1981*a,b*); and linear analogue self-assessment (LASA) (Priestman & Baum, 1976). A QL instrument based upon LASA technique has been developed at this institute and is the subject of a previous report (Selby *et al.*, 1984). This instrument appears to be a valid measure of QL parameters, gives reproducible results and is manageable in the clinical setting.

In the present study we have applied the QL instrument to two groups of patients taking part in a randomized trial of the use of either high dose or low dose chemotherapy for metastatic breast cancer. We have used the instrument to address three questions:

(i) Does the instrument allow discrimination between two groups of patients with similar disease parameters but whose treatment would be expected to elicit quite different levels of toxicity?

(ii) How is patient recall of chemotheray toxicity influenced by the timing of QL assessment in relation to chemotherapy?

(iii) What agreement is there between patient and physician assessment of the same aspect of QL?

Correspondence: D.R. Bell.
Received 5 June 1984 and in revised form 10 December 1984.

*Study design* The QL instrument has been described in detail previously (Selby *et al.*, 1984). It consists of 31 descriptive categories (dimensions) which assess the patient's function and behaviour, features related to breast cancer, and treatment-related effects (Table I). Each dimension is listed above a 10 cm line anchored at its ends by descriptive phrases (Figure 1). The right hand end describes normality or the absence of a symptom and the left hand end the opposite extreme. The patient was asked to mark each linear analogue scale at a point which she felt best described her state for the particular dimension. The instrument can also be used by an independent observer to make an assessment on each dimension for each patient. Patient and observer responses are scored by measuring the distance (in cm) from the left of the line to the mark, giving a range of scores from 0 to 10.

extremely severe vomiting ______________________ no vomiting

**Figure 1** Example of linear analogue scale for the dimension of vomiting.

Twenty-five patients taking part in a prospective, randomized trial comparing low dose CMF (cyclophosphamide 300 mg $M^{-2}$, methotrexate 20 mg $M^{-2}$, fluorouracil 300 mg $M^{-2}$, all given i.v. every 21 days) with high dose CMF (cyclophosphamide 600 mg $M^{-2}$, methotrexate 40 mg $M^{-2}$, fluorouracil 600 mg $M^{-2}$ given by the same schedule) were asked to enter this QL study. Twelve patients received low dose CMF and 13 patients received high dose CMF. All patients had received between 2 and 5 courses of chemotherapy.

Patients were assessed on two occasions (Table II). In this study the independent observer was a physician (D.B.) who was not involved in the clinical management of these patients. The first assessment occurred in the clinic 3 weeks after a course of chemotherapy, just before the

© The Macmillan Press Ltd., 1985

**Table I** Quality of life dimensions

| A. *General-health related dimensions* | B. *Disease related dimensions* |
|---|---|
| 1. Mobility around home, town or country | 1. Pain |
| 2. Regular out-of-home employment | 2. Breathing |
| 3. Self-care | 3. Fatigue |
| 4. Physical activity | 4. Appearance of body |
| 5. Recreation, pastimes or hobbies | 5. Attractiveness to opposite sex |
| 6. Anxiety | 6. Anxiety |
| 7. Social life, meeting and dealing with people outside the family | C. *Treatment related dimensions* |
| 8. Sleep (reduced or increased) | |
| 9. Depression | 1. Nausea |
| 10. Family relationships and marriage | 2. Vomiting |
| 11. Housework | 3. Alopecia |
| 12. Speech | 4. Stomatitis |
| 13. Writing | 5. Dysuria |
| 14. Alertness and mental function | 6. Diarrhoea |
| 15. Anger | 7. Constipation |
| 16. Appetite (reduced or increased) | |

**Table II** Summary of assessment procedure

First Assessment (3 weeks after chemotherapy)
1. Physician assessment by interview in clinic
2. Patient LASA
3. Then chemotherapy administration

Second Assessment (24 h later)
1. Physician assessment by telephone interview
2. Patient LASA

administration of the next course. This is the usual setting in which a physician would assess the toxicity of a particular regimen of chemotherapy. Patients were asked to recall how they had felt in the 24 h following the last course of chemotherapy. The second assessment occurred the following day, 24 h after another identical course of chemotherapy. Each assessment consisted of a standard clinical interview by the physician, who was blinded to the doses of CMF being administered in order to eliminate bias. Following the interview the patient was asked to complete the QL instrument. The physician, using a separate QL instrument, made an assessment of each dimension for that patient, based upon information obtained in the clinical interview. Patient and physician assessments were performed independently and neither had access to the other's assessment. The second assessment, 24 h later, was of similar format except that on this occasion the interview was conducted by telephone. Neither physician nor patient had access to their own previous assessment.

Scores for most items had a unimodal distribution that was skewed toward the end of the scale indicating normality or absence of a symptom. We have used non-parametric methods to analyze our data because of this skewed distribution. The degree of correlation between patient and physician scores was calculated by Spearman's rank correlation. Comparison of scores for each dimension between patients receiving high and low dose chemotherapy was performed using the Wilcoxon rank sum test. Patient groups were comparable for age, prior therapy and distribution of metastatic disease. Thus we would not expect to detect differences in general-health or disease related dimensions between these two-well matched groups. Our analysis has concentrated upon dimensions related to toxicity of treatment.

Median scores and range of scores for patient and physician assessment of dimensions related to treatment toxicity are shown in Table III. Comparison of patient scores from the high dose group with the low dose group, at the first assessment (3 weeks after chemotherapy), showed a statistically significant difference for diarrhoea ($P=0.05$). Differences in scores for alopecia approach significance ($P=0.08$). Comparison of physician scores at the first assessment showed statistically significant differences for alopecia ($P=0.05$) and diarrhoea ($P=0.03$). Thus at the first assessment discrimination between the two groups could be made on the basis of two out of the six toxicity dimensions. Comparison of patient scores obtained at the second assessment (24 h after chemotherapy), showed a statistically significant

**Table III** Median scores and range for patient and physician assessments of toxicity dimensions

| | *First assessment (3 weeks after chemotherapy)* | | | | *Second assessment (24 h after chemotherapy)* | | | |
|---|---|---|---|---|---|---|---|---|
| | *Low dose* | | *High dose* | | *Low dose* | | *High dose* | |
| *Dimension* | *Patient* | *Physician* | *Patient* | *Physician* | *Patient* | *Physician* | *Patient* | *Physician* |
| Nausea | 9.62 (5.3–9.9) | 7.95 (3.0–9.9) | 9.66 (6.6–9.9) | 8.20 (0–9.4) | 8.45 (0.6–9.9) | 8.45 (3.0–9.9) | 7.45 (2.3–9.6) | 6.20 (2.6–9.9) |
| Vomiting | 9.68 (9.5–9.9) | 9.66 (8.1–9.9) | 9.67 (0–9.9) | 9.61 (1.4–9.9) | 9.68 (5.6–9.9) | 9.68 (3.2–9.9) | 7.95 (0–9.9) | 8.12 (1.8–9.9) |
| Alopecia | 9.62 (8.8–9.9) | 9.55 (8.3–9.9) | 9.55 (0.9–9.9) | 7.95 (3.2–9.9) | 9.53 (5.6–9.9) | 9.45 (5.7–9.9) | 5.95 (0–9.9) | 7.95 (2.3–9.9) |
| Stomatitis | 9.68 (7.7–9.9) | 9.68 (8.1–9.9) | 9.68 (9.5–9.9) | 9.68 (9.5–9.9) | 9.67 (6.1–9.9) | 9.72 (9.5–9.9) | 9.68 (8.1–9.9) | 9.68 (8.8–9.9) |
| Dysuria | 9.68 (9.5–9.9) | 9.68 (9.5–9.9) | 9.68 (9.5–9.9) | 9.68 (9.5–9.9) | 9.72 (9.5–9.9) | 9.72 (9.5–9.9) | 9.68 (3.7–9.9) | 9.68 (3.7–9.9) |
| Diarrhoea | 9.68 (9.1–9.9) | 9.68 (9.5–9.9) | 9.62 (5.7–9.9) | 9.66 (5.4–9.9) | 9.72 (9.5–9.9) | 9.72 (9.7–9.9) | 9.59 (5.2–9.9) | 9.59 (7.4–9.9) |

difference between the two groups for vomiting ($P=0.002$) and alopecia ($P=0.04$), while the difference for diarrhoea approached significance ($P=0.08$). When physician scores from this second assessment were compared, statistically significant differences were found for nausea ($P=0.04$), vomiting ($P=0.01$) and diarrhoea ($P=0.05$). At this second assessment discrimination between the high and low dose CMF groups could be made on four of the six toxicity dimensions.

Table IV shows Spearman's rank correlation coefficients for patient and physician scores of toxicity dimensions. Good agreement is seen for most dimensions ($r_s>0.5$). The correlation coefficient was $<0.5$ for dysuria: however, patient and physician scores were high (mean$>9.0$) with small variance, so that substantial agreement is not evident because of poor dispersal of data. There was also good agreement for the general health and disease related dimensions (data not shown). Only

**Table IV** Spearman's rank correlation coefficient of toxicity scores from self-assessment and assessment by a physician

| *Dimension* | $r_s$ | *P* |
|---|---|---|
| Nausea | 0.65 | 0.0001 |
| Vomiting | 0.79 | 0.0001 |
| Alopecia | 0.56 | 0.0003 |
| Stomatitis | 0.69 | 0.0001 |
| Dysuria | 0.47 | 0.0028 |
| Diarrhoea | 0.79 | 0.0001 |

for appearance, relationships, hobbies, anxiety, concentration, anger, breathing and sleep were correlation coefficients less than 0.5. Once again substantial agreement was masked by poor dispersal of the data for concentration and breathing. In general there appears to be good agreement between self-rating and independent observer assessment of the same QL dimension.

Differences in toxicity between high and low dose CMF regimens are to be expected. Our results demonstrate that this QL instrument can discriminate between different levels of treatment related toxicity, even among a small patient sample. We found differences between the patient groups in the first assessment made 3 weeks after chemotherapy for the dimensions of alopecia and diarrhoea; differences in the second assessment at 24 h after chemotherapy were present for the dimensions of nausea, vomiting, alopecia, and diarrhoea. It seems likely that patient recall has underestimated toxicity experienced 3 weeks previously. The toxicities of alopecia and diarrhoea may be more lingering and thus more easily recalled than the more acute and short-lived toxicities of nausea and vomiting. There appears to be better recall of acute toxicity when QL assessment is made closer to the time chemotherapy administration.

Currently the evaluation of this QL instrument shows that there are minor differences in discriminative capacity when the instrument is used for self-rating compared with its use by an independent observer. Although the good correlation between patient and physician scores indicate that they were both assessing similar aspects of QL, the minor discriminative differences suggests that the most valuable QL information may come from combining self-rating with assessment by an independent observer. Currently a larger study is underway at this institution: its aims are to determine whether or not differences may exist in other dimensions between groups of patients with breast cancer, to assess change in LASA with time, and to correlate change in LASA with response to chemotherspy.

Quality of life assessment should become an integral part of all clinical trials that seek to assess the impact of treatment on palliation. The present instrument has potential for routine clinical use. It can be used readily by patients themselves as well as by independent observers, and we have shown evidence of its discriminative capacity in this small group of patients. Information on QL is critical in the determination of the overall effectiveness of chemotherapy regimens and in the comparison of different regimens.

### References

BERGNER, M., BOBBIT, R.A., CARTER, W.B. & GIBSON, B.S. (1981). The sickness impact profile: Development and final revision of a health status measure. *Med. Care,* **19,** 787.

PRIESTMAN, T.J. & BAUM, M. (1976). Evaluation of quality of life in patients receiving treatment for advanced breast cancer. *Lancet,* **i,** 899.

SELBY, P.J., CAMPBELL, J. ETAZADI-AMOLI, J., DALLEY, D. & BOYD, N.F. (1984). Evaluation of a method of assessing the quality of life in patients with metastatic breast cancer. *Br. J. Cancer,* **50,** 13.

SPITZER, W.D., DOBSON, A.J., HALL, J. & 5 others. (1981). Measuring the quality of life of cancer patients. *J. Chron. Dis.,* **34,** 585.